# Altered Interoceptive Predictive Coding in Functional Neurological Disorders

**DOI:** 10.1111/psyp.70417

**Published:** 2026-10-07

**Authors:** Natascha Stoffel, Michaël Mouthon, Andria Pelentritou, Laure von der Weid, Cristina Concetti, Marzia De Lucia, Selma Aybek

**Affiliations:** ^1^ Department of Neurology, Faculty of Science and Medicine University of Fribourg Fribourg Switzerland; ^2^ Graduate School for Health Sciences (GHS) University of Bern Bern Switzerland; ^3^ Brain‐Body and Consciousness Laboratory, Department of Clinical Neuroscience Lausanne University Hospital, University of Lausanne Lausanne Switzerland; ^4^ Center for Biomedical Imaging Lausanne Switzerland

**Keywords:** conversion disorder, EEG, heartbeat‐evoked potentials, Interoception, predictive coding

## Abstract

Interoception, the processing of internal bodily signals, has been linked to the pathophysiology of Functional Neurological Disorder (FND), along with the theoretical concept of altered predictive coding (PC). FND symptoms such as sensory‐motor dysfunction may arise from disrupted integration of sensory input and predictions. However, PC in the modality of interoception has not yet been investigated in FND. Using a PC paradigm, the cardio‐audio synchronization task measured electrophysiological responses to the heartbeat during omitted sounds that were temporally locked to the heartbeat (synchronous condition) or not (asynchronous control condition). Patients with mixed FND symptoms (*N* = 36) were compared to sex‐ and age‐matched healthy controls (HC: *N* = 42) by contrasting synchronous and asynchronous heartbeat‐evoked potentials during omissions using cluster‐based permutation statistics. We further tested associations with self‐reported interoceptive variables and assessed whole‐scalp main and interaction effects in global field power (GFP) and topography. Patients with FND showed no cluster‐level differences between conditions, whereas we identified a central cluster difference in HCs. The interoceptive‐specific contrast (synch‐asynch difference waves) differed between groups and correlated with interoceptive self‐report but only when tested across groups (*r* = −0.33, *p* = 0.012). In this paradigm, group‐by‐condition interactions in topography identified the insula as the most probable source of differences. These interactions occurred before those identified in GFP analyses, suggesting that differential networks emerge first, followed by changes in overall activity strength. These results indicate that patients with FND use different neural mechanisms for interoceptive PC (i.e., precision‐weighted prediction errors in interoception‐guided predictions) than HCs. Patients with FND showed no difference in interoceptive PC, suggesting a dysfunctional interoceptive system. Together, these findings suggest that interoceptive‐cued regularity processing in FND is characterized by altered early network recruitment, followed by differences in overall signal strength, potentially reflecting a disrupted progression of *interoceptive PC*.

## Abstract 2

This study makes use of the cardio‐audio synchronization task which aims to measure interoceptive predictive coding in Functional Neurological Disorder and their sex‐aged healthy controls. We demonstrate altered neural responses with no differentiation for interoceptive‐cued omissions compared to their control condition in FND, unlike the healthy control cohort. Interaction effects are estimated to originate in insular deactivation, providing novel evidence of aberrant interoceptive function in FND.

## 1 Introduction

Functional neurological disorders (FND) involve neurological symptoms including motor dysfunction (e.g., paralysis, tremor, or dystonia), sensory dysfunction, dizziness, or dissociative/functional seizures, arising from altered brain network function (Hallett 2022). This disorder challenges the traditional, dualistic understanding of the body and mind, as it arises at the interface between the psyche and the soma, within the brain's signal integration (Aybek and Perez 2022). Over the past decades, research has elucidated several associated mechanisms, highlighting the importance of predictive coding (PC), salience processing and salience circuits, and interoception (Drane et al. 2021). Interoception refers to the processing of internally generated signals, providing a moment‐by‐moment mapping of the body's internal landscape (Khalsa and Lapidus 2016). PC frameworks have been used to model how information about the “world” is continuously updated in the nervous system by incorporating new information and reducing redundancy. Under such PC frameworks, model updating occurs through the transmission of unexpected or unpredicted inputs, commonly referred to as prediction errors (Huang and Rao 2011; Seth et al. 2012). From the outside, the “world” comprises exteroceptive signals, unifying visual or tactile inputs for example, while from the inside, the “world” is described by interoception, unifying signals such as those from the cardiovascular system (Quigley et al. 2021; Tsay et al. 2015). While the processing of external signals has been widely studied, interoception has only recently gained attention in neuroscientific and clinical research. Importantly, growing evidence suggests that interoception is implicated in a variety of clinical conditions that might also implicate FND (Khalsa and Lapidus 2016; Nord and Garfinkel 2022).

In FND, recent evidence shows that patients have lower interoceptive accuracy, as measured by heartbeat counting or detection tasks (Koreki et al. 2020; Ricciardi et al. 2016; Williams et al. 2021), while other studies produce conflicting results (Koreki et al. 2023; Millman et al. 2023; Sojka et al. 2021). In addition, interoceptive and salience networks comprising regions such as the insula and cingulate cortices appear less activated or co‐activated in FND patients than in controls, with insular activation associated with interoceptive accuracy (Sojka et al. 2025). A recent analysis of the same FND cohort as studied here, demonstrated interoceptive dysfunction in the respiratory domain, highlighting less efficient interoceptive sensory accumulation (Stoffel, Sojka, et al. 2025). The same population showed attenuated heartbeat‐evoked potentials (HEP; the neurophysiological response to the heartbeat) at specific frontal‐lateralized sites, demonstrating interoceptive dysfunction at a neuronal level with an objective marker (Stoffel, Mouthon, et al. 2026). Two other, independent studies in a specific subgroup of FND, namely patients with functional/dissociative seizures, have before linked this attenuation of the HEP to the onset of functional/dissociative seizures, when compared to epileptic seizures or to their own baseline (Elkommos et al. 2023; Flasbeck et al. 2024). Symptom generation in FND may be explained by such a PC framework (Edwards et al. 2012; Hallett et al. 2022; Jungilligens and Perez 2024), in particular within altered interoceptive processing that might precede or be involved in symptom formation (Flasbeck et al. 2024). The PC framework has scarcely been tested empirically, with—to our knowledge—only one study examining exteroceptive sensory processing and thereby showing apparent abnormalities in a patient cohort with FND (Matthews et al. 2020). In the interoceptive modality, the amplitude of the HEP has been proposed to be a measure of the precision‐weighted prediction error of the ongoing heartbeat. Thus, increased HEP amplitudes, which result from directing attention toward the heartbeat, may represent the relative precision associated with each heartbeat (Ainley et al. 2016; Petzschner et al. 2019). These HEP markers, already found to be lower in FND as a trait (Stoffel, Mouthon, et al. 2026) and as a state (Elkommos et al. 2023; Flasbeck et al. 2024), should now be further investigated within a PC paradigm.

The brain constantly encodes and integrates different signals, extracting regularities between them to construct internal models of events and their relationships (Seth et al. 2012). This occurs across interoceptive and exteroceptive domains: the brain can extract temporal regularities from interoceptive inputs like the heartbeat and use them to predict future events for the sense of “presence”. Exteroceptive inputs like auditory stimuli are used to predict future events for the sense of “agency” (Seth et al. 2012). Pfeiffer and De Lucia (2017) developed a task to test whether PC may involve both interoceptive and exteroceptive signals. Using an auditory oddball paradigm, in which regularities across internal cardiac and external auditory stimuli were established, they tested what we herein refer to as *interoceptive PC*. This *cardio‐audio synchronization task* (CAS) measured how interoceptive signals such as the heartbeat can facilitate processing of external auditory regularities (Pfeiffer and De Lucia 2017). This paradigm tests whether the brain uses interoceptive inputs from the ongoing heartbeat as a cue to predict an exteroceptive input, such as a sound. Using the CAS, an auditory stimulus is presented in synchrony with the heartbeat, measuring the surprise response at the neuronal level when the expected sound is omitted. The control condition is generated by presenting the same auditory stimuli asynchronously, with sounds delivered based on previously recorded R‐peak‐to‐R‐peak intervals. Therefore, the control condition is a sequence of sound‐to‐sound intervals matched to the synchronous condition, but without any relationship to the ongoing cardiac rhythm because the R‐peak‐to‐sound intervals are variable. Previous literature has shown differences between the synchronous and asynchronous conditions in healthy volunteers, suggesting that the healthy brain uses the heartbeat as a temporal cue to form expectations about future auditory events (Pelentritou et al. 2024; Pfeiffer and De Lucia 2017).

Here, to address the question of whether *interoceptive PC* is different in FND compared to healthy controls (HC), we tested whether HEPs upon omitted sounds were different between the two populations and between the synchronous and asynchronous conditions, when an association between heartbeat and sound was formed (synchronous condition) or not (asynchronous control condition). We were interested in *interoceptive PC*, herein referring to how interoceptive signals, specifically the heartbeat, would aid the prediction of external sounds. We thus measured HEP when the sound was unexpectedly omitted in a condition where the sound was expected in time with the heartbeat (synchronous), compared with a condition where no such expectation tied to the heartbeat exists (asynchronous). To the best of our knowledge, this is the first time such a task like the CAS task has been used to infer the degree of *interoceptive PC* in patients with FND. We contrasted their HEP responses during omissions with those of a cohort of HCs matched for age and sex. This study empirically tested the previously hypothesized PC account of FND's pathophysiology, focusing on the modality of interoception. We broadly hypothesized that: (I) Unlike HCs, patients with FND will lack the expected difference in the degree of surprise upon omission between the synchronous and the asynchronous control condition. (II) Compared to HCs, patients with FND would show less activation in cortical areas implicated in sensory or interoceptive processing, measured via altered source‐level cortical activations in response to the omitted sound from the sound sequence in synchrony with the heartbeat. (III) The neuronal markers derived from differences in group or condition, or their interaction, would correlate with other interoceptive measures derived from the heartbeat counting task and self‐reported questionnaire scores, as well as clinical symptom variables such as examiner‐rated symptom severity scores or self‐rated occurrence of somatoform dissociation symptoms.

## 2 Methodology

### 2.1 Participants

This study was a part of a larger study with 92 enrolled FND and HC participants that assessed biological markers (Stoffel, Ansede‐Bermejo, et al. 2026; Stoffel, von der Weid, et al. 2025) and interoceptive function (Stoffel, Sojka, et al. 2025; Stoffel, Mouthon, et al. 2026). Patients were recruited from the Cantonal Hospital in Fribourg (HFR) and the Psychosomatic Medicine Unit at Inselspital Bern, while controls were recruited via flyers and word‐of‐mouth. Inclusion criteria for patients were an official FND diagnosis (DSM‐5/ICD‐11), and for patients and controls were an age ≥ 18 and a capacity to consent. Exclusion criteria included major psychiatric or neurological comorbidities, brain surgery or implants, substance abuse, cardiovascular disease, inability to follow procedures, and pregnancy or breastfeeding. The study was approved by the Ethics Committee of Canton Bern (2023‐00469), registered at clinicaltrials.gov (NCT06084325), and conducted in accordance with the Declaration of Helsinki. All participants gave written informed consent.

Of the 92 participants recruited for the larger interoception study, *N* = 14 had to be excluded from the analyses presented here. These exclusions consisted of *N*_FND_ = 4 due to high symptom severity, which prevented data acquisition during an approximately three‐hour study visit. The overall study visit described in detail in Stoffel, von der Weid, et al. (2025) included various parts and other interoceptive tasks (see: Stoffel, Mouthon, et al. 2026; Stoffel, Sojka, et al. 2025) before the CAS paradigm was performed at the end. *N*_FND_ = 2 and *N*_HC_ = 1 were excluded due to a large number of excluded epochs (> 33% of epochs), indicating poor EEG quality. *N*_FND_ = 1 and *N*_HC_ = 3 were excluded due to large differences (> 30 ms) in the interstimulus interval between the synchronous and asynchronous conditions, indicating a poor match to the cardiac rhythm in the asynchronous condition. *N*_HC_ = 2 were excluded due to technical issues with real‐time R‐peak detection, and *N*_FND_ = 1 was excluded for falling asleep during the task. In summary, with *N* = 78 as the final sample size, 85.87% of the original, full population was successfully measured and reported here.

The final cohort included in the CAS study consisted of patients diagnosed with FND (*N* = 36, 75% female, mean age = 38.6 years with SD = 11.6) and HC (*N* = 42, 76.2% female, mean age = 37.2 years with SD = 12.5), matched for sex and age to the FND population.

### 2.2 Psychological Assessment

Participants completed the MAIA (Mehling et al. 2012) and IAS (Murphy et al. 2020) questionnaires one to three days before the experiment to assess interoceptive self‐report. On the day of the experiment, participants also completed the SDQ‐20 (Nijenhuis et al. 1996), BDI‐II (Beck et al. 1996), and STAI (Spielberger et al. 1970), and provided information on medication use, comorbidities, and, for females, menstrual cycle or hormonal contraception. Patients underwent a neurological assessment by the examiner to rate the clinical global impression (CGI; Guy 1976) and obtain scores on the short form of the movement disorder rating scale (SFMDRS; Nielsen et al. 2017) in FND patients with motor symptoms (*N* = 26).

### 2.3 Interoceptive Predictive Coding Task

Participants completed the CAS paradigm, an auditory oddball paradigm linked to the heartbeat, in accordance with (Pfeiffer and De Lucia 2017). Participants were not informed about the experimental manipulation, which involved administering sound either in synchrony (synch) or asynchrony (asynch) with the ongoing heartbeat. They were instructed to listen passively to the sounds, while seated still, with eyes open and fixating on a screen. In a preceding task lasting 20 min, we recorded participants' EEG and ECG to measure the HEPs (Stoffel, Mouthon, et al. 2026). During this time, an algorithm was trained to detect R‐peaks in real time and to record each participant's heart rhythm (see the Supporting Information for details). During the CAS paradigm, real‐time detection was used to position the stimuli in synchronization with the participants' heartbeats (synch condition), with a mean R‐peak‐to‐sound delay of 48 ms (SD = 8 ms). For the asynch condition, the participants' previously recorded interbeat intervals (R‐peak to R‐peak) were shuffled to determine the timing of the stimuli (asynch condition). Stimuli were either a sound (a 1000 Hz sine wave sampled at 44.1 kHz, with a duration of 100 and 70 ms of ramp‐up and ramp‐down) played via in‐ear headphones at a comfortable volume, or an omission (no sound). The sequence of sounds (80% of stimuli) and omissions (20% of stimuli) was randomized, ensuring at least one sound between two omissions, with both the first and last stimulus in each block being a sound (mean number of sounds between two omissions: 3.97 ± 3.88). The CAS experiment consisted of four blocks, each containing 300 stimuli and lasting approximately 5 min, presented in a pseudo‐randomized order: either asynch‐synch‐synch‐asynch or synch‐asynch‐asynch‐synch. At the end of the task, participants were asked whether they perceived a difference between the four blocks, whether they formed an idea of what the task was about, and what the sounds represented. These control questions confirmed that all participants were blind to the task's purpose.

### 2.4 Behavioral Interoceptive Measurements

As part of a larger study on interoception, we also assessed behavioral interoception (cardiac using the heartbeat counting task, and respiratory using the respiratory resistance sensitivity task), which are introduced in the Supporting Information and discussed in detail in previous work (Stoffel, von der Weid, et al. 2025).

### 2.5 EEG and ECG

EEG was recorded at 2048 Hz using a 64‐channel BioSemi ActiveTwo system, referenced to the CMS‐DRL ground, placed occipitally (BioSemi, Amsterdam, Netherlands). ECG was recorded via four electrodes placed diagonally across the torso—two below the clavicles and two at the lower rib cage. The signal from the right clavicle to the left rib cage was used for R‐peak detection in 95.5% of participants; the other diagonal pair served as a backup and was used for the remaining 4.5% of participants, for whom this pair provided a better signal.

#### 2.5.1 EEG Preprocessing

EEG data were pre‐processed offline using a custom MATLAB toolbox (EEGpal, available on https://github.com/DePrettoM/EEGpal) based on EEGLab 2025.0 (Delorme and Makeig 2004). Raw EEG data were bandpass filtered (0.3–40 Hz; Widmann et al. 2015) and cleaned using ICA with ICLabel (Pion‐Tonachini et al. 2019) on concatenated files per participant to remove ocular and cardiac artifacts. To identify and remove cardiac field artifacts, the procedure described by Buot et al. (2021) and adapted by Cataldi et al. (2025, 2026) was implemented. Mean pairwise phase consistency (PPC) was computed between the ECG signal and each independent component (IC) across frequencies from 0 to 25 Hz, for our data using epochs ranging from −100 to 500 ms relative to the R‐peak (Vinck et al. 2010). ICs exhibiting PPC values exceeding three standard deviations above the mean were flagged as potential cardiac artifact components and subsequently subjected to visual inspection. Components were classified as cardiac artifacts based on the presence of characteristic features, including left–right asymmetry in scalp topography and/or a sharp, time‐locked deflection coinciding with ECG R‐peaks throughout the recording. We then removed identified cardiac artifact components from the EEG data. On average, 2.44 ± 0.77 components were removed per participant. Bad channels were visually identified (Brunet et al. 2011) and interpolated using spherical splines (Perrin et al. 1989), with 1.22 electrodes interpolated on average (1.96% ± 1.8%). Data were re‐referenced to the common average. No baseline correction was implemented. HEPs were computed for each condition by averaging epochs from −100 to 500 ms relative to the R‐peak, following Pfeiffer and De Lucia (2017). Only HEPs during sound omissions were included in the analysis. For the asynch condition, epochs from −100 to 500 ms relative to the R‐peaks closest to the omission were chosen, while controlling that the chosen R‐peak was at least 500 ms after the preceding sound. Finally, noisy epochs with a maximal absolute amplitude higher than 100 μV were excluded. On average, 97.93% of epochs were retained as artifact‐free. For further analysis, a grand average per group and per condition was calculated for the −100 to 500 ms around the R‐peak during the omission blocks.

### 2.6 Statistical Analysis

For behavioral data, group differences were calculated using *t*‐tests for numeric normally distributed data and Wilcoxon tests for non‐normally distributed data. Chi‐squared tests were used for binary variables. Correlation analyses were performed using Pearson correlations, and Cohen's *d* was used for the calculation of effect sizes. The *R* software (version 4.3.3) was used for behavioral statistical analyses.

For EEG omission HEPs, the statistical analysis was divided into two parts: (i) a spatio‐temporal cluster permutation statistical analysis exploring differences between conditions (synch vs. asynch) as well as between group and condition (FND vs. HC for synch minus asynch) using Fieldtrip (Oostenveld et al. 2011), and (ii) a global analysis, exploring overall differences in topography or global field power (GFP) with a 2 × 2 factor design using the Ragu toolbox (Koenig et al. 2011). In addition, we identified the sources of the observed group differences in the global analysis using the sLORETA algorithm (Pascual‐Marqui 2002). For both analyses, a minimum duration of significant differences was set at 10 ms.

#### 2.6.1 EEG Cluster Analysis

For the EEG cluster analysis, single‐subject omission HEPs in the synch and asynch conditions were compared separately within each group using cluster‐based permutation paired *t*‐tests (Maris and Oostenveld 2007). This approach enabled the identification of spatially and temporally resolved differences in neurophysiological activity between the two conditions, while accounting for multiple comparisons across electrodes and timepoints. Secondly, differences in EEG amplitude between the two conditions were investigated by subtracting the asynch from the synch epochs (synch‐asynch) to assess differences between groups using cluster‐based permutation unpaired *t*‐tests. In all cases, 10,000 permutations were performed, and clusters with a Monte Carlo *p*‐value < 0.05 (two‐tailed) were considered statistically significant. This approach was chosen to isolate condition‐dependent omission HEP modulation within each group and then test whether the magnitude of this modulation differed between groups. Rather than testing a Group × Condition interaction, this approach focuses on the effect itself, operationalized as the synch–asynch difference wave, which can reduce potential confounds arising from baseline group differences in overall EEG amplitude.

Correlation analyses were conducted to determine the association between neurophysiological variables of interoceptive processing and other dimensions of interoceptive processing, or clinically relevant variables. This was done by extracting the mean amplitude of the identified clusters in the time interval where the synch‐asynch contrast was significant between the two groups and correlating the mean amplitude with the following variables of interest: MAIA, IAS, respiratory sensitivity, and heartbeat count task accuracy. The analysis was performed across groups to test the interoceptive variables as a continuum, but when correlations were significant, it was repeated separately for each group to test for its presence despite the underlying group differences in these variables. Significant correlations of *p* < 0.05 are reported herein, alongside raw *p*‐values and *p*‐values after correcting for multiple comparisons using false discovery rate (FDR).

#### 2.6.2 Global EEG Analysis

For the global EEG analysis (ii), the 2 × 2 factor design (group: FND vs. HC × condition: synch vs. asynch) was used to extract overall differences in topographical patterns (using topographical ANOVA; TANOVA) and signal strength (using GFP) between omission HEPs using the Ragu toolbox (Koenig et al. 2011), performing assumption‐free randomization statistics. Here, we report the main effects of group and condition, as well as their interaction. For the TANOVA analysis, we applied L2 normalization to ensure detected topographic differences were not due to overall differences in signal strength. The *p*‐threshold was set at < 0.05, and the analyses were computed with 5000 randomizations to correct for multiple comparisons. The Cartool software (Brunet et al. 2011) was used to visualize differences between GFPs. Note that, for whole‐scalp analyses, no covariate control was conducted due to methodological constraints. However, we performed the same analyses on the medication‐free cohort to partially address the potential effect of medication on the observed differences in omission HEPs.

Because we were also interested in the neuronal networks underlying the identified whole‐scalp interactions (in either TANOVA or GFP), we assessed the most probable source for the time intervals that showed interaction effects in the global analyses. This test visualizes the identified interaction intervals, rather than serving as a separate, independent test for source localization. This source localization was performed using voxel‐wise independent *t*‐tests (FND vs. HC) and permutation testing (5000 randomizations) on sLORETA‐derived current density maps (Pascual‐Marqui 2002), using untransformed data with a signal‐to‐noise ratio (SNR) of 100, subject‐wise scaling, and no baseline correction. The results of this analysis are presented as the most probable source of averaged time intervals of the identified TANOVA or GFP group differences, represented by three‐dimensional statistical maps with t‐values across cortical voxels. Note that source localization served as a visualization tool for the networks involved in the identified interaction effects, rather than representing a separate statistical test.

#### 2.6.3 Control Analysis

We performed a series of control analyses to control for factors that could affect interpretation of the results. First, we looked at heart rate (HR) and heart rate variability (HRV) between FND and HC and between the synch and asynch conditions. Second, we assessed the intervals between the R‐peak and sound across conditions and populations to ensure the experimental paradigm was correctly implemented. Third, the interval between sounds was computed to ensure comparability across experimental conditions. For these control analyses, repeated‐measurement ANOVAs were performed with condition as a within‐subject factor and group as a between‐subject factor. Statistical results of post hoc tests were corrected for multiple comparisons using the Tukey method.

In addition, to ensure that differences in the cardiac signal did not influence the observed omission HEP results, we performed three direct comparisons (*t*‐tests) on individual averages of epoched ECG signals (−100 to 500 ms relative to the R‐peaks closest to the omission), correcting the statistic for the number of points tested (FDR). First, we checked for differences in condition (synch vs. asynch) separately within each group, then compared the synch‐asynch subtraction for FND versus HC.

Finally, due to the potential effects of medication (psychotropic, opioid, benzodiazepine, and anti‐epileptic medication) on brain activity, we repeated the global EEG analysis after excluding all participants who reported taking such medication. These control analyses are presented in the Supporting Information.

## 3 Results

### 3.1 Clinical Characteristics

The two groups in our study population did not differ in terms of sex or age (*p* > 0.6). Yet, patients with FND exhibited a higher score of depression (*t* = 5.94, *p* < 0.001) and anxiety (state: *t* = 3.45, *p* < 0.001 and trait: *t* = 4.26, *p* < 0.001). Due to the high correlation of trait anxiety and depression scores (*r* = 0.80, *p* < 0.001), a sum score of these two variables was created to add to the model as a covariate for affective symptoms. Also, patients with FND reported a higher intake of psychotropic, antiepileptic, opioid, and benzodiazepine medications (*χ* ^2^ = 16.84, *p* < 0.001). Interoceptive self‐report was measured using two different questionnaires, in which patients with FND scored lower (Table 1, MAIA: *d =* −0.77, *p* = 0.001 and IAS: *d =* −0.64, *p* = 0.036). Tables S1, S2, and S4 in the Supporting Information provide more details on clinical comorbidities, FND symptom subtypes, and subgroup demographics without medication.

**TABLE 1:** Demographics of healthy controls (HC) and patients with functional neurological disorder (FND).

	HC	FND	*p*
	*N* = 42	*N* = 36	
Sex (count female)	32.0 (76.2)	27.0 (75.0)	> 0.9^a^
Age (mean)	37.2 (12.5)	38.6 (11.6)	0.6^b^
Medication (psychotropic, antiepileptic, opioids, benzos)	2.0 (4.8)	17.0 (47.2)	< 0.001^a^
Body mass index (median)	22.7 (3.5)	25.0 (5.5)	0.2^c^
Heart variability: RMSSD (median)	40.2 (37.1)	29.5 (18.9)	0.007^c^
RR‐interval (mean)	911.8 (122.3)	838.6 (105.0)	0.006^b^
Depression: BDI‐II (median)	3.0 (6.0)	15.0 (14.0)	< 0.001^c^
State anxiety: STAI‐S (median)	28.5 (9.5)	38.0 (21.3)	< 0.001^c^
Trait anxiety: STAI‐T (mean)	37.4 (10.2)	46.1 (12.4)	< 0.001^b^
Trait interoceptive awareness: MAIA_TOTAL_ (mean)	24.0 (4.5)	19.7 (6.5)	0.001^b^
Trait interoceptive accuracy: IAS (mean)	89.0 (14.3)	80.0 (27.0)	0.043^c^

^a^ Chisq‐test (count, %).

^b^ *t*‐test (mean, SD).

^c^ Wilcoxon‐test (median, IQR).

### 3.2 EEG Cluster Differences in Interoceptive PC

#### 3.2.1 Differences Between Groups

The cluster analysis served to identify differences between omission HEPs in the synch compared to the asynch condition, separately for HC and FND patients. For HCs, this analysis revealed differences in amplitude between the two conditions (*p* = 0.002), with higher amplitude for the asynch compared to the synch condition in central electrodes (mainly FC1, C1, C2, CP1 and CPz) between 120 and 323 ms following the R‐peak (Figure 1a). For the FND group, we did not observe a main effect of condition (*p* > 0.05).

**FIGURE 1:**
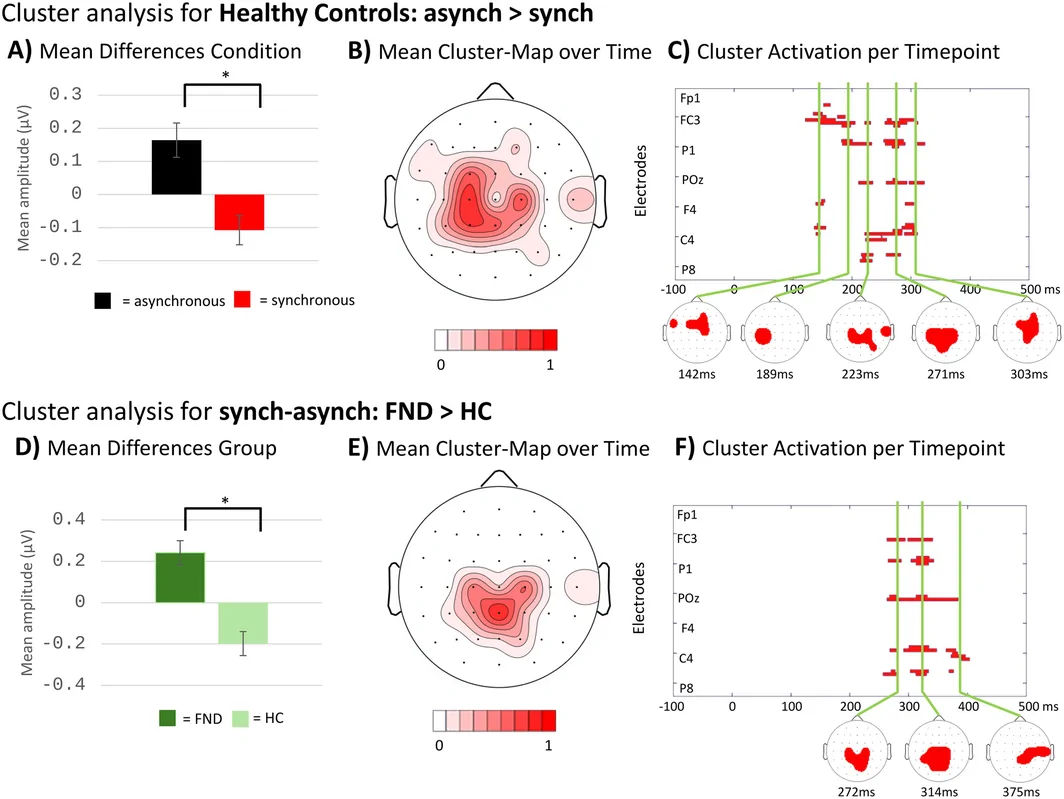
Visualization of results on cluster analyses. (A) Shows the mean differences in μV for the condition effect seen in the Healthy Controls with whiskers representing the standard error (B) Illustration of the Topography of the Cluster activated for this condition effect over the full‐time span with red electrodes being activated longer and (C) shows the Cluster Activation per timepoint with individual electrodes activation pattern shown per specific time windows. (D) Shows the mean difference in μV for the group effect for the contrast of condition (synch‐asynch) with whiskers representing the standard error (E) Illustration of the Topography of the Cluster activated for this group effect over the full‐time span with red electrodes being activated longer and (F) shows the Cluster Activation per timepoint with individual electrode activation pattern shown per specific time windows.

#### 3.2.2 Synch‐Asynch Cluster Differences Between Group

To test cluster group differences between HC and FND cohorts, the EEG signal from the asynch condition was subtracted from the synch condition (synch‐asynch), and this difference was then tested between groups using cluster‐based permutation statistics. This analysis identified a positive cluster for FND compared to HC (*p* = 0.040) at centro‐posterior electrodes (mainly C1, Cz, C2, CP1, CPz, Pz and P2) between 257 and 403 ms following the R‐peak (Figure 1B). Extracting the mean amplitudes of this significant cluster within the identified time interval, the Cohen's *d* effect size was 1.21, and linear mixed models also confirmed that the group difference would remain when adding covariates such as medication intake, affective symptoms, or cardiac differences (*p* < 0.001). Using these extracted synch‐asynch contrast amplitudes per participant further allowed us to test correlations between these neural activation patterns and other interoceptive variables. A significant correlation across groups was identified for interoceptive self‐report, such as the IAS *r* = −0.33 with *p* = 0.003 (FDR‐adjusted to *p* = 0.012) and the MAIA_TOTAL_ *r* = −0.25 with *p* = 0.025 (FDR‐adjusted to *p* = 0.057). However, repeating this correlation separately for each group, these correlations were not significant for either group (IAS: *p* > 0.083 and MAIA_TOTAL_: *p* > 0.545).

### 3.3 EEG Global Differences in Interoceptive PC

#### 3.3.1 Main Effects of Condition and Group

Investigating the global group differences in topography (TANOVA) and overall signal strength (GFP) in FND patients and HCs using two‐factor ANOVA, a main effect of condition was observed in the TANOVA between 56–72 ms (*p* = 0.008), 144–167 ms (*p* = 0.006), and 382–410 ms (*p* = 0.030) following the R‐peak (Figure 2A). Further, a main effect of condition was detected for GFP at 31–55 ms (*p* = 0.005), 77–96 ms (*p* = 0.020), 247–261 ms (*p* = 0.018), and 476–488 ms (*p* = 0.008) following the R‐peak (Figure 2B).

**FIGURE 2:**
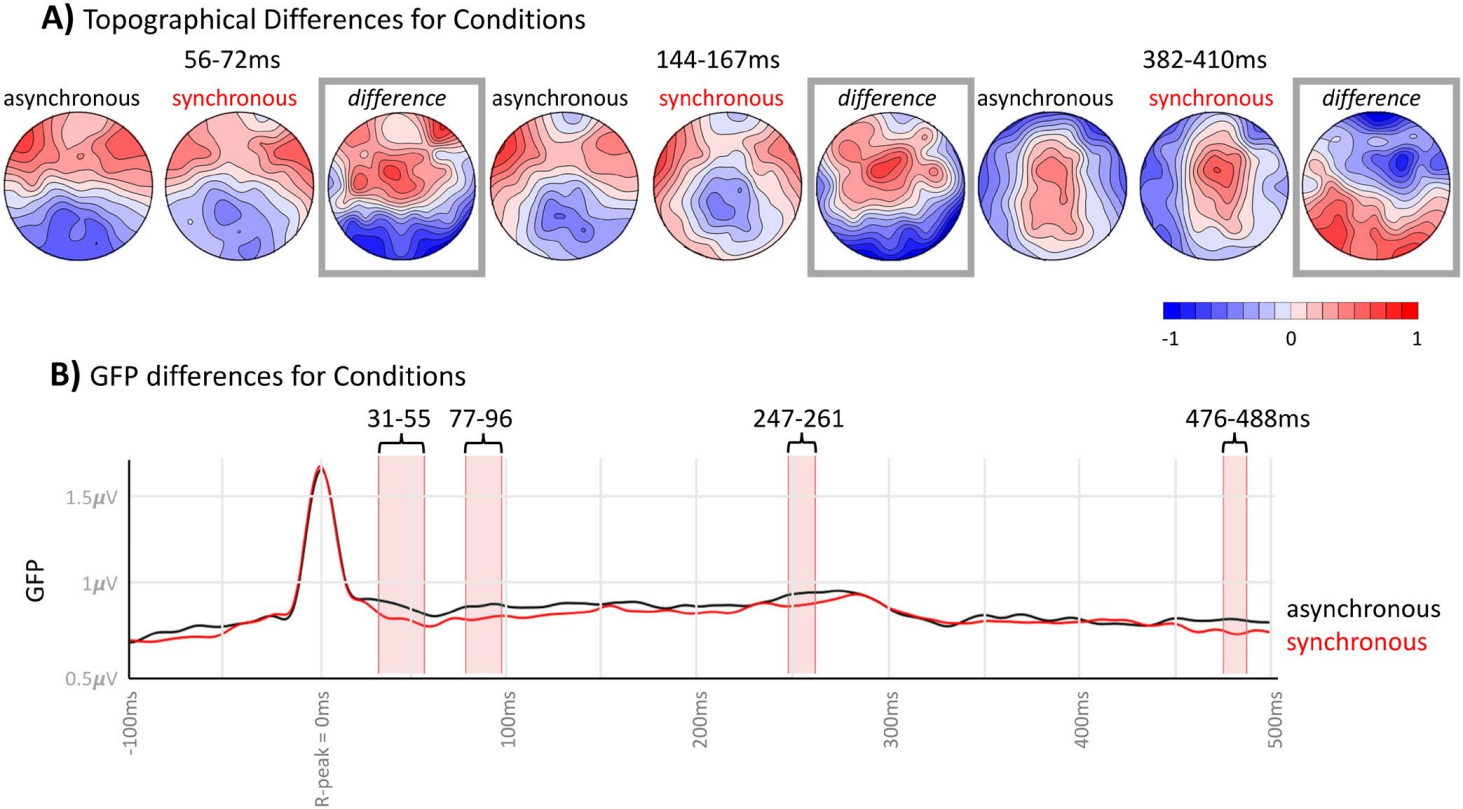
Topographical differences (TANOVA) and global field power (GFP) for the omission heartbeat evoked potentials in the synchronous and asynchronous conditions independent of cohort. (A) Shows the associated topographies in the time interval of significant differences along with the difference map to highlight the changes between the conditions. Please note that due to the L2 normalization before the GFP analysis, the colorbars represent the change from −1 to 1 (which is not in μV) (B) shows the time intervals with a significant difference in GFP. Significance portrays *p* < 0.05, computed from the randomization analysis.

Finally, the TANOVA identified a main effect of group at 463–480 ms (*p* = 0.020) and for GFP at 10–20 ms after the R‐peak, with higher GFP in patients with FND than in HC (*p* = 0.005).

#### 3.3.2 Interaction of Group and Condition

For topographical differences identified using a TANOVA, we observed an interaction effect between condition and group just before the R‐peak between −17 and −5 ms (*p* = 0.005), as well as at 181–197 ms (*p* = 0.020), 215–241 (*p* = 0.020), and 309–322 ms (*p* = 0.030) after the R‐peak (Figure 3A). The sources of these topographical differences were visualized by calculating the inverse solution of the contrast (synch‐asynch) between groups using sLORETA. Using this approach, the insular region was identified as the most probable source of this interaction effect, with less insular activation at earlier time intervals for the condition contrast in the FND cohort than in HCs (Figure 4). Note that for visualization, the two time intervals that were temporally close (181–197 and 215–241 ms) were merged. In the interaction visualization (FNDsynch‐FNDasynch vs. HCsynch‐HCasynch), the negative *t*‐statistics for the contrasts indicate a smaller difference between the conditions in insular activation. That means that for HCs, the contrast between the synch and asynch conditions stems from altered insular activation, the hub for interoceptive processing. And this was not the case for our FND cohort. Finally, for the later time interval of significant interaction (i.e., 309–322 ms), peak detection identified Brodmann area 45 in the inferior frontal gyrus and frontal lobe as the most probable source for the identified topographical difference.

**FIGURE 3:**
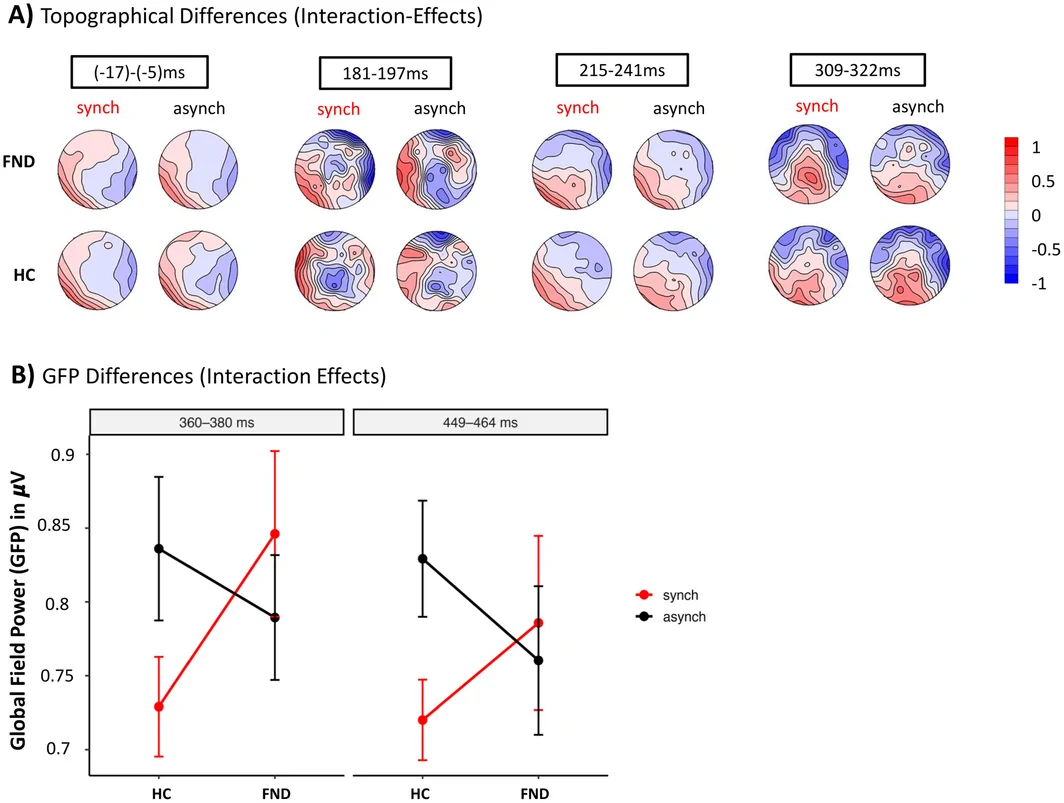
Demonstration of interaction of group and condition. (A) Differences in Topography (TANOVA), illustrated separately per group and condition for each time interval with a significant interaction effect. For further analyses on the source of these topographical interactions, please see Supporting Information that contain estimated source localization for the group contrasts, identifying a difference in insular activation for the first three time intervals. (B) Global field power (GFP) illustrating mean GFP plotted for each group (FND and HC) across the identified time interval. Points represent condition‐wise group means; error bars indicate 95% bootstrapped confidence intervals. Lines connect mean values across groups within each condition. Note that all time intervals were derived from running a 2 × 2 factor model with 5000 permutations.

**FIGURE 4:**
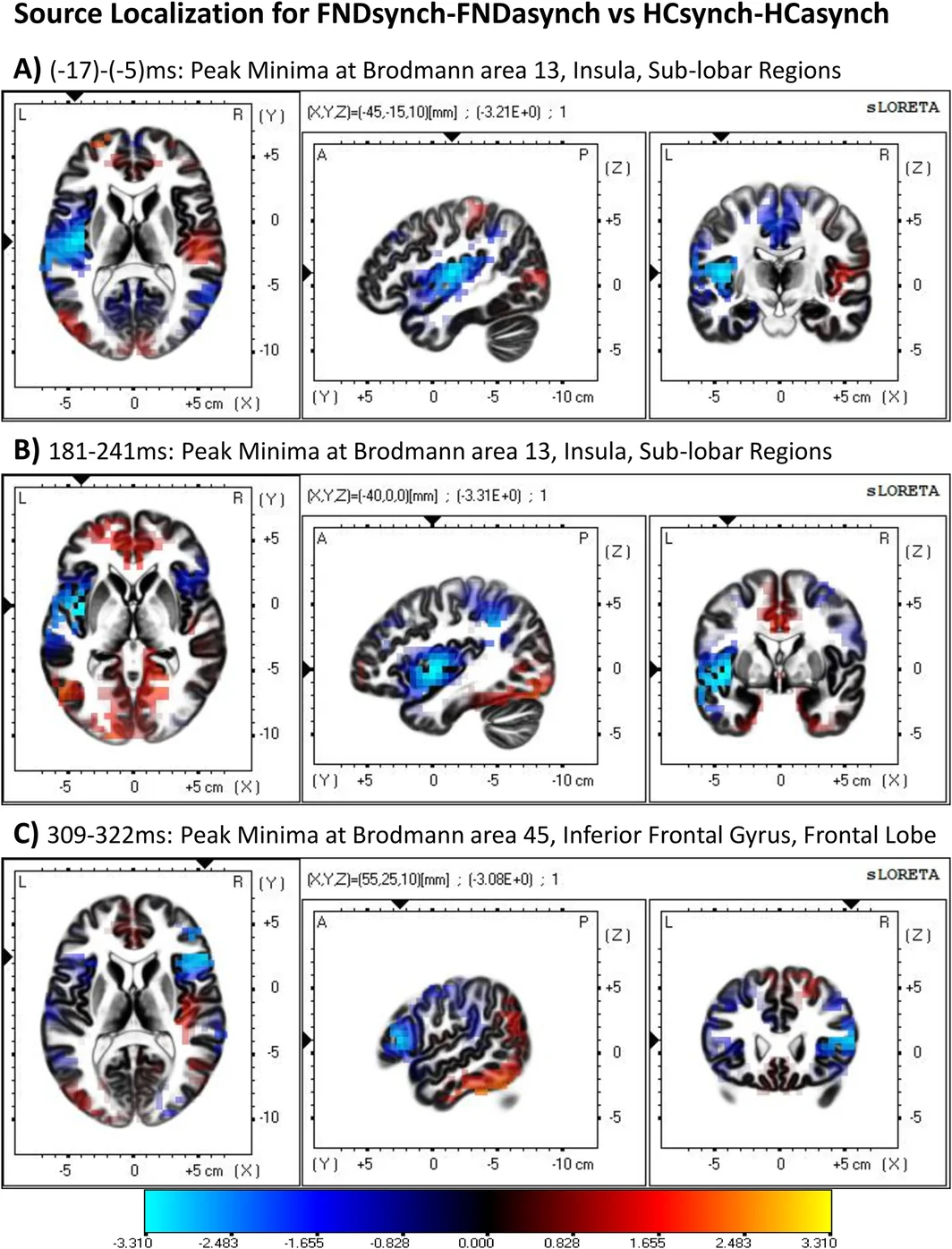
Visualization of origin for the three intervals of topographical differences from interaction‐effects, using an independent *t*‐test in sLORETA with dependent variables (FNDsynch‐FNDasynch vs. HCsynch‐HCasynch). Thus, the identified regions for the source localization shows the activation for FND compared to HC for a contrast of synch‐asynch with peak minima localized at Brodmann area 13, Insula and sublobar regions for (A) just before the heartbeat and (B) 181–241 following the R‐peak, and Brodmann area 45, Inferior Frontal Gyrus, Frontal Lobe for (C) at 309–322 ms following the R‐peak.

For GFP, there was also an interaction effect between condition and group at 360–380 ms (*p* = 0.020) and at 449–464 ms (*p* = 0.013) after R‐peak (Figure 3B). The source of these GFP differences was visualized using sLORETA for both conditions and groups, identifying a peak minimum for the group comparison of this contrast for the time interval 360–380 ms following R‐peak in frontal regions, as well as a peak maximum for the time interval 449–465 following the R‐peak at temporal regions (Figure S1).

### 3.4 Control Analysis

We performed a series of control analyses to check if the observed results were trivially driven by the task design. For each analysis, we used a repeated‐measures ANOVA to examine the effects of group (FND, HC) and experimental condition (synch, asynch). First, we analyzed the R‐peak‐to‐R‐peak intervals in terms of HR and HRV. We observed differences in HR and HRV between the two groups (FND vs. HC), with *F*(1,76) = 7.86, *p* = 0.006, and *F*(1,76) = 13.00, *p* < 0.001, respectively. Importantly, there were no differences between the experimental conditions (synch vs. asynch): *F*(1,76) = 0.66, *p* = 0.420 (Figure 5A,B). For the second control analysis, we tested the variability in the delay between the R‐peak and sound (R‐peak‐to‐sound interval). As expected, there was a difference between the synch and asynch conditions (*F*(1,76) = 3398.6, *p* < 0.001), with an additional between‐group difference (*F*(1,76) = 7.44, *p* = 0.008), mainly in the asynch condition (post hoc *t*(76) = −2.27, *p*_Tukey_ = 0.035) (Figure 5C). Next, we tested variability in the time delay between two sounds (sound‐to‐sound interval). Also as expected, given the underlying differences in HR and HRV, this variability differed between the two groups (FND vs. HC): *F*(1,76) = 8.46, *p* = 0.005. However, the analysis also showed a significant difference between the synch and asynch conditions (*F*(1,76) = 15.64, *p* < 0.001), driven by the HC group (post hoc *t*(76) = 3.73, *p*_Tukey_ = 0.002) (Figure 5D).

**FIGURE 5:**
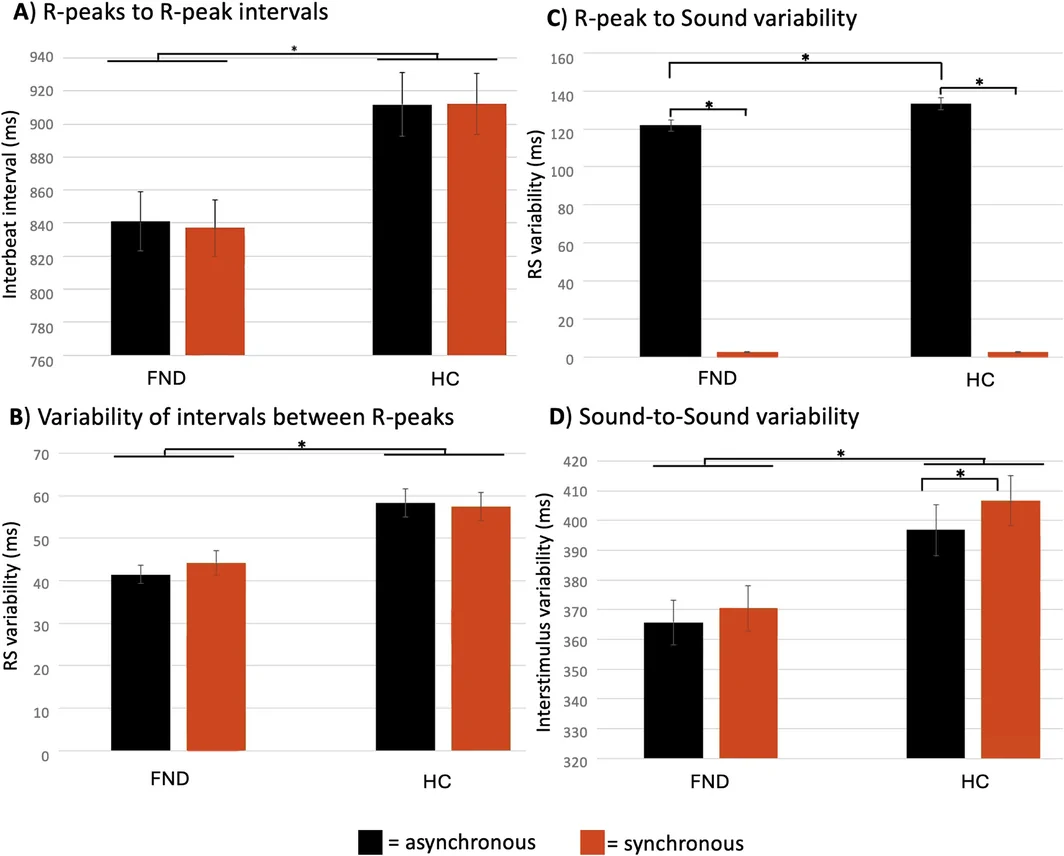
Control analysis for the CAS paradigm in FND patients and HCs: Interbeat intervals for the two populations separately, illustrating (A) the mean and (B) its standard deviation. (C) Standard deviation of the absolute interval between the R‐peak and the sound onset. (D) Standard deviation of the sound‐to‐sound interval. *p < 0.05.

No significant differences were found in the ECG signal between the synch and the asynch conditions for either FND or HC over the period of interest (*p* > 0.05 with FDR correction, Figure S2a,b). Similarly, the comparison between groups for the difference ECG wave of synch‐asynch did not show a significant difference (Figure S2c).

Finally, in the medication‐free population (*N* = 59, including *N* = 19 patients with FND), the central EEG cluster findings of group differences for the synch‐asynch contrast were not replicated. For the global whole‐scalp analyses, however, the results were partially replicated. For the condition effects, the first two of the three significant TANOVA time intervals remained significant in the medication‐free subsample. For the GFP condition effects, three of four were the same, with three additional time intervals newly identified as significant. For the main effect of group, no significant intervals were identified. Finally, for the interaction effect using the TANOVA, the 182–197 ms interval remained significant, along with a novel earlier (72–87 ms) and later (390–408 ms) interval. For the GFP, both intervals were observed in the medication‐free subsample (364–381 and 452–464 ms) (for an overview of the results from the full sample compared to the medication‐free sample regarding the whole‐scalp analysis, see Table S4).

## 4 Discussion

The CAS task examined perception of *interoceptive PC* by assessing the brain's response to heartbeats that were temporally linked to omitted sounds in sequences that were either synchronous or asynchronous with the heartbeat. We identified a central cluster for a within‐group difference in HCs, suggesting that only healthy controls inferred the relationship between heartbeat and sound. This condition effect was absent for patients with FND, indicating a dysfunctional interoceptive process due to this absence of differences between the synchronous and asynchronous conditions. We also observed a significant central cluster when comparing the synch‐asynch contrast between conditions in HCs versus FND patients (Figure 1). At a global level, there were main effects of condition on both topography and overall activity (Figure 2). Similar time intervals to those observed in the cluster differences at 257–403 ms following the R‐peak (i.e., for the condition contrast between groups using synch‐asynch for FND vs. HC), were identified as an interaction effect when whole‐scalp analyses were performed using a 2 × 2 factor design for both the TANOVA (i.e., 215–241 ms following R‐peak) and the GFP (i.e., 360–380 ms following R‐peak) (Figure 3). These findings suggest that FND is associated with altered *interoceptive PC*, demonstrated by the absence of differential neuronal processing of interoceptive signals compared to exteroceptive signals in predicting co‐occurring stimuli. Finally, this is further supported by identifying altered activation in the insular region during identified interaction intervals (Figure 4).

### 4.1 Interoceptive‐Exteroceptive Binding

Cluster analysis revealed altered neural mechanisms in patients with FND for integrating interoceptive cues to predict external auditory stimuli, compared to sex‐ and age‐matched HCs. When contrasting the synchronous and asynchronous omission‐HEPs for HCs, we observed a central negative cluster at 120–323 ms following the R‐peak during the omitted sound; however, this comparison was not significant for FND patients (see Figure 1). A positive peak around 300 ms following a stimulus typically represents the ERP component P3. This component has been associated with stimulus evaluation and discrimination, selective attention, and is typically amplified by unexpected or salient stimuli (Patel and Azzam 2005; Squires et al. 1977). The earlier component of stimulus evaluation and discrimination, particularly in auditory oddball paradigms, is known as the Mismatch‐Negativity response (MMN): a fronto‐central negativity to the difference between deviant and standard stimuli, occurring ~100–250 ms after stimulus onset (Garrido et al. 2009; Patel and Azzam 2005). We interpret the negative cluster in healthy controls as an MMN response when contrasting the synchronous and asynchronous conditions, consistent with previous findings using the CAS paradigm (Pelentritou et al. 2024). These findings suggest that HCs encoded the regularity established across cardiac and auditory signals and were surprised when omission violated this regularity, inducing an MMN response. This response is absent in the FND cohort. The absence of this characteristic ERP in the FND cohort supports the idea that FND patients may have a dysfunctional interoceptive system or predictive coding compared to HCs (Lyndon 2026). Patients with FND show no difference between the conditions, indicating that they do not form predictions based on the cardio‐audio coupling. Importantly, exteroceptive and interoceptive cues usually interact, demonstrating that our interoceptive system dynamically adapts to the external world. This interactive adaptation seems necessary for stable bodily self‐processing (Engelen et al. 2023; Park and Blanke 2019a) and might be altered in FND.

Enhanced interoceptive processing may stem from top‐down regulation, modulated by explicit cues (Marshall et al. 2017). Thus, variation in omission‐HEPs across conditions may reflect interoceptive learning that minimizes prediction errors (Marshall et al. 2017; van Elk et al. 2014). This understanding may explain the findings in the HC and their absence in the FND cohort. Owens et al.'s (2018) discussion supports this idea, proposing that a failure of top‐down precision control within interoceptive systems leads to impaired attentional regulation of ascending bodily signals. This is consistent with the altered predictive coding account suggested for FND's underlying mechanisms (Lyndon 2026). Such dysfunction could reduce the ability to flexibly disengage from internal cues or predictions. Applying this interpretation to our EEG data suggests that patients with FND do not exhibit differential neurophysiological activity in central clusters, arguably as a P3 component, for synchronous omissions (i.e., showing no difference between synchronous and asynchronous conditions). This may be because they have not formed reliable *predictions* about co‐occurring auditory events and cannot differentially attenuate their own interoceptive responses. These results thus support the theory of disrupted *interoceptive PC* in FND, involving altered brain activation in interoception‐related tasks (Flasbeck et al. 2024; Sojka et al. 2025).

### 4.2 Interoceptive‐Specific Central Clusters Associated With Interoceptive Self‐Report

When comparing the interoceptive contrast between groups (i.e., asynchronous subtracted from the synchronous HEP during the omission), we identified a significant central‐parietal cluster difference (involving mainly EEG channels C1, CP1, Pz, CPz, Cz, C2, CP2, P2) between 257 and 403 ms following the R‐peak. This central cluster, identified as a group difference, also correlated with self‐reported interoceptive accuracy (IAS) across groups. However, this correlation was significant only when these interoceptive variables were tested as a continuum in a pooled group analysis, which conflates within‐ and between‐group variance and may yield significant effects due to underlying group differences. These findings therefore can only partly suggest a link between differential processing of the interoceptive system using neural markers and self‐reported interoceptive accuracy scores. Some of the previous investigations of interoception in FND have reported correlations between self‐reported interoception and clinical characteristics such as seizure frequency (Koreki et al. 2020), symptom severity (Millman et al. 2023), and dissociation scores (Yogarajah et al. 2019). However, as the group difference within the central cluster across groups also disappeared when only medication‐free patients were included (see Supporting Information for a detailed discussion of the medication‐free subsample analysis), we must also highlight the need for caution when interpreting these results, as they may be due to an underlying group effect, the effects of medication, or driven by existing comorbidities.

### 4.3 Overall Main Effects for *Interoceptive PC*

At the global level, and as a main effect of condition, we identified differences in topography using TANOVA at 144–167 ms, indicating differences in the activated neuronal networks for each condition. We also identified differences in GFP at 247–261 ms, indicating overall differences in signal strength between the synchronous and asynchronous conditions (see Figure 2). These results support those previously reported by Pfeiffer and De Lucia (2017) in healthy volunteers, who observed an effect of global map dissimilarity at 158–190 ms and of GFP at 240–271 ms. Notably, our results seem also primarily driven by the HC group. Additional effects to those of Pfeiffer and De Lucia (2017) emerged in our pooled cohort at earlier (56–72 ms) and later (382–410 ms) time windows for TANOVA and at 31–55, 77–96, 459–466, and 476–488 ms for GFP; however, these were also predominantly driven by the HC group.

We also identified a main effect of group between patients and HCs in GFP at 10–20 ms around the R‐peak and in TANOVA at 463–480 ms following the R‐peak. These effects were independent of the auditory condition (i.e., whether the sounds were played in synchrony or asynchrony with the heartbeat beforehand). Therefore, this neurophysiological activity may suggest an anticipatory or preparatory response to the heartbeat itself, as well as distinct topographies at later time points. However, note that these findings should also be treated with caution, as the group comparison was not corrected for covariates and the identified time intervals are quite short.

### 4.4 Altered *Interoceptive PC* in FND

Finally, also at the whole‐scalp level, we identified various group‐by‐condition interactions, suggesting an overall altered *interoceptive PC* in FND compared to HCs (see Figure 3). Using a TANOVA, we identified four distinct early interaction intervals, suggesting differences in the topography of the group‐by‐condition interaction. One interval occurred before the R‐peak (−17 to –5 ms), indicating a potential difference in anticipation of heartbeat‐linked sounds. This may be connected to the previously discussed main effect of group. The other topographical interaction time intervals all appeared within the first 322 ms post R‐peak, suggesting that early differences between groups and conditions in the CAS paradigm may be related to the recruitment of different neural sources and networks. In addition to the above, the interaction effects identified in GFP, which indicate differences in overall signal strength, were significant only in later time windows (starting at 360 ms post R‐peak). These later intervals may also reflect general HEP effects (Park and Blanke 2019b). We want to point out again, that within the same cohort, an attenuated HEP and the involvement of attentional guiding have already been identified (Stoffel, Mouthon, et al. 2026). However, the time windows in which these intervals were detected for the GFP condition‐by‐group interaction were similar to those observed in the EEG cluster analyses. As noted above, these findings may indicate a “surprise” response to violated predictions stemming from comparing conditions within the HC and FND groups separately. The results of this GFP interaction indicate that HCs can flexibly use heartbeat‐cued expectations for temporal expectations (i.e., showing an attenuation of overall GFP activation in response to omissions of synchronous sounds compared to asynchronous sounds), whereas patients with FND exhibit indifferent neuronal activation in response to omitted sounds, which may or may not be linked to their heartbeat. Indeed, 300 ms after the R‐peak, two interaction effects were observed in the GFP analysis. While HCs exhibited stronger overall activation in the asynchronous condition, FND patients showed stronger activation in the synchronous condition. This extends the findings of group‐specific EEG differences following cluster permutation analysis and supports further dysfunctional interoceptive activation in FND (Flasbeck et al. 2024; Sojka et al. 2025).

The topographical differences suggest distinct activation of neural networks involving regions such as the insula in the synchronous condition compared with the asynchronous condition (see Figure 4). The reduced GFP in the synchronous condition suggests lower overall processing demands for stimuli that are predicted by interoception. The insula has been found to be more active in interoceptive rather than exteroceptive recall (DeVille et al. 2018), and has been shown to be less active in FND cohorts than HCs during interoceptive tasks (Sojka et al. 2025). This also supports the view that, in healthy individuals, the process of interoception may be continuous and efficient, recruiting insular regions (DeVille et al. 2018), and aligns with the notion of heartbeat‐related sensory suppression, whereby internally predicted signals elicit early and automatic attenuated responses (van Elk et al. 2014).

However, these findings may represent group differences that are not only attributable to altered *interoceptive PC*, but may also be partially driven by affective symptoms or medications distributed unevenly across the FND and HC groups. Nevertheless, as most of these global results remain significant even when all participants taking medication are excluded (see Supporting Information for a detailed description), we are confident that these findings reveal important FND characteristics within the interoceptive system.

### 4.5 Limitations

A noteworthy limitation of this work relates to the different intervals and timings between the sounds in the asynch and synch conditions. As outlined above, the algorithm used for real‐time R‐peak detection was trained on data acquired during a task in which participants were instructed to focus on either their heartbeat or an external auditory stimulus (white noise). At the end of each 20‐s focusing trial, participants responded to questions about their internal associations during the attention period. While this procedure effectively maintained task engagement and attentional focus, it introduces a methodological limitation in the experimental paradigm described herein. Specifically, the algorithm was trained on the entire trial duration, including not only the passive focusing phase but also the subsequent active response phase. This set‐up may have introduced notable variability in heart rate attributable to task‐related processes such as movement initiation or cognitive appraisal. Such task‐induced fluctuations in cardiac rhythm, arising from the transition between passive attention and active response phases, may have influenced the heart rate patterns used to construct the asynchronous conditions. As a result, the sound‐to‐sound intervals in these conditions did not precisely match those observed in the synchronous conditions; an effect reflected in increased variability of sound‐to‐sound intervals in the asynchronous compared to the synchronous condition.

In addition, cardiac artifacts may confound the omission HEP comparisons (e.g., see Steinfath et al. 2025). While cardiac artifacts were removed using rigorous methods as part of our preprocessing pipeline, we cannot rule out that any effects appearing around the R‐peak may be driven by residual cardiac field artifacts. However, because we performed within‐group and between‐group analyses targeting differences by condition or group, we note that these cardiac artifacts would be comparable across the synch and asynch conditions. Indeed, a control analysis contrasting ECGs within the synch and asynch conditions separately per group, as well as between groups, revealed no differences in electrical cardiac activity.

Another considerable limitation is that the interpretation of our results relied on the assumption that the heartbeat was temporally locked to the sound. While these R‐peak‐to‐sound delays are comparable to previous literature using the CAS paradigm (Pelentritou et al. 2024, 2025; Pfeiffer and De Lucia 2017), we would still like to address the approximate delay of 43.5 ms which may not be interpreted as being simultaneous (Pollatos et al. 2014). This delay reflects a fixed temporal offset that was fairly constant (SD = 5.5 ms). However, it remains unclear at what stage of the cardiac cycle the human may process the heartbeat (e.g., within the systolic vs. diastolic phase). Hence, while we interpret our synch condition as (perceived) synchronous with the heartbeat, with a fixed R‐peak‐to‐sound delay, this limitation should still be kept in mind.

We also want to raise the issue of multiple statistical tests and the risk of reporting false positives. Because this task is novel in the clinical population of FND, our analyses were exploratory (hence the use of different approaches and the consideration of short time intervals of at least 10 ms as significant). In addition, we did not perform rigorous covariate‐control analyses for EEG. Nonetheless, the sub‐sample analysis on the medication‐free subset of patients and controls showed similar interaction patterns; however, it did not replicate the main effect of group, suggesting that medication might play a role in some of our findings. Similarly, additional comorbidities and other covariates may have influenced the global analyses, as this analysis (implemented as a 2 × 2 factor model) could not control for covariates.

Another limitation is that the CAS paradigm was the last task after approximately 2 h of additional experimental paradigms' administration, including different interoceptive assessments. This led to participants (especially patients) becoming fatigued by the time the CAS paradigm was administered. In addition, participants may have been implicitly biased toward interoceptive processing from previous tasks (Chen et al. 2024).

Finally, the study did not assess self‐reported symptom severity; instead, it derived it from external examination. Subjective ratings of quality of life or symptom severity would better reflect the burden to participants and their beliefs, which may correlate more with the mechanisms investigated using the CAS paradigm, as measured here.

## 5 Conclusion

Our study revealed differences in how patients with FND predict and process internal bodily signals compared to a healthy control group. Patients with FND showed no difference in omission‐HEP when comparing sounds displayed in synchrony with their heartbeat to the control condition with asynchronous heartbeat‐sound coupling. This was evident when using both a condition contrast in cluster‐based permutation statistics and whole‐scalp 2 × 2 factor analyses. Conversely, in the HC cohort, we found higher omission amplitudes during the asynchronous condition than the synchronous condition in a central cluster. These results suggest that patients with FND may struggle to predict external events (e.g., sounds) based on internal signals (e.g., heartbeats), whereas HCs can engage in differential interoceptive learning to make precise predictions using these cues. Furthermore, topographical EEG analyses revealed altered brain activity in regions associated with interoceptive processing, such as the insula. This was identified as the source of interaction effects between group and condition. Finally, the central cluster identified as a group difference for the condition contrast (synch‐asynch) correlated with self‐reported scores on the interoceptive accuracy scale, the IAS, but only across groups and not when tested separately within groups. This highlights the need to further investigate the congruence of neural markers and subjective reports in the interoceptive modality. In summary, our results demonstrate that patients with FND exhibit altered *interoceptive PC* compared to HC. This intriguing finding may indicate that integrating internal body signals differentially from external events is related to a stable sense of bodily ownership, perception, agency, and self‐consciousness (Aspell et al. 2013; Heydrich et al. 2018; Suzuki et al. 2013). Dysfunctional integration of internal and external inputs may lead to symptoms such as uncontrollable movements, not‐perceived touch, or even dissociation (Lyndon 2026). Therefore, altered *interoceptive PC* (i.e., indifferent processing of internal signals in relation to synchronized external events) may underlie FND symptomatology. While this study reveals novel and interesting results, they should be interpreted as preliminary and in requirement of further replication that might also address some of the raised limitations. Our findings emphasize the central role of disrupted interoceptive processing in FND and suggest that specific neural responses to learned regularities across bodily signals and external sensory inputs may be promising targets for future diagnostic and therapeutic strategies for patients with FND.

## Author Contributions

**Natascha Stoffel:** conceptualization, methodology, investigation, data curation, formal analysis, visualization, writing – original draft, project administration, writing – review and editing. **Michaël Mouthon:** methodology, validation, investigation, data curation, formal analysis, writing – review and editing. **Andria Pelentritou:** validation, writing – original draft, writing – review and editing. **Laure von der Weid:** investigation, writing – review and editing. **Cristina Concetti:** writing – review and editing. **Marzia De Lucia:** supervision, writing – review and editing. **Selma Aybek:** supervision, writing – review and editing, funding acquisition.

## Funding

This work was supported by the Swiss National Research Foundation Professorship (Grant PP00P3_176985).

## Conflicts of Interest

The authors declare no conflicts of interest.

## Supporting information


**Table S1:** Clinical details and comorbidities.
**Table S2:** FND symptom subtypes.
**Figure S1:** Visualization of origin for GFP differences from interaction‐effects, using the epochs of both conditions and group and calculating an independent *t*‐test with dependent conditions in sLORETA (FNDsynch‐FNDasynch vs. HCsynch‐HCasynch). Thus, the identified regions for the source localization shows the activation for FND compared to HC for this contrast of synch‐asynch with a peak minima at Brodmann area 46, the Middle Frontal Gyrus and the Frontal Lobe for (A) at 360–380 ms following the R‐peak, and a peak maxima at Brodmann area 39, the Middle Temporal Gyrus and the Temporal Lobe at (B) for 449–465 ms following the R‐peak.
**Figure S2:** Differences of the ECG during omission trials between conditions and groups (same analysis as in Figure 1 of main manuscript). (A) FND = patients with functional neurological disorders and (B) HC = healthy controls comparison comparing synchronous (red) and asynchronous (black) using a paired *t*‐test. (C) Same analysis using the difference wave of synch‐asynch traces of the HEP during omissions of sound to compare FND and HC ECG traces. No significance detected.
**Table S3:** Demographics for subpopulation after exclusion of medicated participants.
**Table S4:** Comparison of the results of the whole‐scalp analyses for the full study population and the medication‐free subsample.

## Data Availability

Experimental script, data, and code that support the findings of this study are openly available at https://github.com/FND‐ResearchGroup/CAS_FND_NS.
