# Supplementary material for: Altered Interoceptive Predictive Coding in Functional Neurological Disorders
Source: Psychophysiology. 2026 Oct 7;63(10):e70417. doi: 10.1111/psyp.70417 (PMC13644901; doi:10.1111/psyp.70417)
Supplement: Supplementary file 1 [file PSYP-63-e70417-s001.docx]

# **Supplementary Material**

# **Altered interoceptive predictive coding in Functional Neurological Disorders**

Natascha Stoffel^1,2^, Michaël Mouthon^1^, Andria Pelentritou^3^, Laure von der Weid^1^, Cristina Concetti^1^, Marzia De Lucia^3,4^, Selma Aybek^1^

**Author affiliations:**

1 Faculty of Science and Medicine, Department of Neurology, University of Fribourg, 1700 Fribourg, Switzerland

2 Graduate School for Health Sciences (GHS), University of Bern, 3012 Bern, Switzerland

3 Brain-Body and Consciousness Laboratory, Department of Clinical Neuroscience, Lausanne University Hospital, University of Lausanne, Lausanne 1011, Switzerland

4 Center for Biomedical Imaging, Lausanne 1011, Switzerland

**Clinical Details of Population**

| **Table S1. Clinical Details and Comorbidities** | | | | |
| --- | --- | --- | --- | --- |
| **Variable** | **Overall** **N = 78** | **HC N = 42** | **FND** **N = 36** | ***p-value*** |
| Intake of Pain Medication [yes] | 13 (17%) | 2 (5%) | 11 (31%) | *0.005* |
| Intake of Psychotropic Medication [yes] | 16 (21%) | 2 (5%) | 14 (39%) | *<0.001* |
| Intake of Sleep Medication [yes] | 7 (9%) | 0 (0%) | 7 (19%) | *0.001* |
| Intake of Other Medication [yes] | 18 (23%) | 6 (14%) | 12 (33%) | *0.065* |
| Comorbidity of ADS or AD(H)D [yes] | 5 (6%) | 1 (2%) | 4 (11%) | *0.2* |
| Comorbidity of Psychiatric Disorder [yes] | 11 (14%) | 2 (5%) | 9 (25%) | *0.010* |
| Comorbidity of Hormonal Dysfunction [yes] | 3 (4%) | 1 (2%) | 2 (6%) | *0.6* |
| Comorbidity of other Diagnosis [yes] | 10 (13%) | 3 (7%) | 7 (19%) | *0.10* |
| Note: statistical test between the two groups, portraying count (%) with Fisher test | | | | |

| **Table S2. FND symptom subtypes** | | | |
| --- | --- | --- | --- |
| **Variable** | **Overall** **N = 36** | **Female N = 27** | **Male** **N = 9** |
| Functional Dissociative Seizures [yes] | 6 (17%) | 6 (22%) | 0 (0%) |
| motor + symptoms [yes] | 13 (36%) | 9 (33%) | 4 (44%) |
| motor - symptoms [yes] | 26 (72%) | 18 (67%) | 8 (89%) |
| sensory symptoms [yes] | 19 (53%) | 15 (56%) | 4 (44%) |
| dizziness (PPPD) [yes] | 2 (6%) | 2 (7%) | **0 (0**%**)** |
| cognitive symptoms [yes] | 3 (8%) | 3 (11%) | 0 (0%) |
| Note: FND symptom frequency where one patient might also report more than one symptom subtype. Numbers indicate count (%). | | | |

## **Description of the real-time R-peak detection algorithm**

Real-time R-peak detection from the continuous ECG was performed using a custom script based on the methodology of Pfeiffer and De Lucia (Pfeiffer & De Lucia, 2017). It was programmed directly in the EEG/EMG acquisition tool ActiView version 903 (Biosemi, Amsterdam, Netherlands) based on LabVIEW 2022 Q3 software (National Instruments, Austin, USA). For each data packet received (on average every 2-4ms), the software calculated the variance over the last 50ms of the ECG signal. If this variance reached the participant's individual threshold (see below for its calculation), a marker was added to the recording (R-peak detected) and the experimental stimulation (either a tone or an omission) was triggered in the synchronous condition. After a hit, there is a cooldown period of 200ms during which no tests were performed to avoid overdetection. We observed a delay of 5.02ms (SD=2.48ms) between real-time and offline R-peak detection. In addition, we measured a sound emission latency of 38.48ms (SD=3.02ms) using a Blackbox Toolkit V2 (The Black Box Toolkit Limited, Sheffield, UK). In summary, the average delay between an R-peak and sound perception was 43.5ms (SD=5.5ms).

An individual R-peak detection threshold was calculated based on the 15-minute ECG signal recorded prior to the main experiment, during which participants were instructed to direct their attention to the heartbeat or an external sound while seated to measure their attentional HEP, similar to the set-up of Petzschner et al (2019) (Petzschner et al., 2019). The script to compute the individual threshold was created in MATLAB R2021b (MathWorks Inc., Massachusetts, USA). First, R-peaks of this recording were detected offline using the script by Sergey Chernenko, available on the website <http://www.librow.com/articles/article-13> (Cherneko, 2007). We checked and confirmed its reliability on our data through visual inspection. A copy of this detection was recorded to define the participant’s cardiac pattern, which was then used in the asynchronous condition of the CAS paradigm. We then calculated the mean variance over 50ms before the R-peaks, present in the first half of the ECG signal, to obtain the first version of the threshold. To optimize this value, different versions of the threshold were used to simulate real-time R-peak detection (30, 40, ..., 100% of the original value), using the same method described in the previous paragraph, over the second half of the ECG signal. The chosen threshold value was the one that produced an average peak position closest to that obtained offline (i.e., the more reliable method). The better of the two available ECG recordings (left or right electrodes at the bottom of the rib cage versus right or left electrodes on the clavicles) was used. This was determined automatically by selecting the channel with the largest mean R-peak amplitudes.

To compare the asynchronous condition with the synchronous condition, we also tested whether heart rate and heart rate variability differed between the more active HEP task and the more passive CAS task. Using a paired Wilcoxon or t-test, separated by group, we did not find a significant difference in these cardiac measures between the two tasks (p > 0.454).

**Further Assessment of Interoceptive Variables**

In the heartbeat-counting task (HCT), participants were instructed to count only the heartbeats they perceived across three time epochs (25, 35, 45s in randomized order). This was repeated once in an arousal condition, where participants were instructed to hold their breath for as long as they could tolerate. The mean of the six trials per person was used as an interoceptive HCT accuracy score.

Participants also completed the respiratory resistance sensitivity task (RRST), a respiratory task using signal detection theory techniques in psychophysics to reliably measure individual sensitivity. After 50 trials, participants showed an individual threshold for detecting obstruction in breathing, which is considered the respiratory sensitivity score. For more details, see previous publication (Stoffel et al., 2025).

**Inverse Solution of Interaction (time intervals significant for the GFP analysis)**

**Figure S1**. Visualization of origin for GFP differences from Interaction-Effects, using the epochs of both conditions and group and calculating an independent t-test with dependent conditions in sLORETA (FNDsynch-FNDasynch vs HCsynch-HCasynch). Thus, the identified regions for the source localization shows the activation for FND compared to HC for this contrast of synch-asynch with a peak minima at Brodmann area 46, the Middle Frontal Gyrus and the Frontal Lobe for A) at 360-380ms following the R-peak, and a peak maxima at Brodmann area 39, the Middle Temporal Gyrus and the Temporal Lobe at B) for 449-465ms following the R-peak

**Control Analysis on the ECG trace**

As a control analysis, to determine whether the detected EEG differences were related to cardiac differences detectable in the ECG trace, the same analysis as for results presented in section 3.2.1 and 3.2.2 was conducted on the ECG trace itself. The ECG signal was filtered between 0.5 and 40 Hz to correct for signal drift and high-frequency noise (Rangayyan, 2001), and averaged using epochs identical to the analyzed HEPs (-100 to 500ms from R-peak).

No significant differences were found between synchronous and asynchronous conditions for either FND or HC (p > 0.05 with FDR correction over the number of time points tested), and the group comparison of the synch-asynch difference waves also showed no significant difference (**Figure S4**). These statistics were computed using the Statistics module of EEGpal (<https://github.com/DePrettoM/EEGpal>), which relies on the Statistics and Machine Learning Toolbox from MATLAB, and the script *fdr_bh* of David Groppe for the FDR correction (https://www.mathworks.com/matlabcentral/fileexchange/27418-fdr_bh).

**Figure S2**. **Differences of the ECG during omission trials between conditions and groups (same analysis as in Figure 1 of main manuscript).** **A**) FND = patients with functional neurological disorders and **B**) HC = healthy controls comparison comparing synchronous (red) and asynchronous (black) using a paired t-test. **C**) Same analysis using the difference wave of synch-asynch traces of the HEP during omissions of sound to compare FND and HC ECG traces. No significance detected.

**Subgroup analysis of non-medicated participants**

The control analysis for the medication effect was done by excluding all participants who reported an intake of psychotropic, antiepileptic, opioid, or benzodiazepine medication. Thus, the sub-analysis included N = 59 participants, of whom N = 19 (32.2%) were patients with FND. Sex and age did not differ between groups in this sub-analysis. By excluding patients with an intake of medication, we arguably also excluded those with high psychiatric comorbidity and thus did not have a significant difference between the groups in state or trait anxiety in this subsample (State: p = 0.057, Trait: p = 0.084). **Table S3**.

| **Table S3: Demographics for subpopulation after exclusion of medicated participants** | | | | |
| --- | --- | --- | --- | --- |
|  | **Overall** **N = 59** | **HC** **N = 40** | **FND** **N = 19** | **p-value** |
| Sex (female) | 43.0 (72.9) | 30.0 (75.0) | 13.0 (68.4) | *0.8 ^a^* |
| Age (mean) | 37.0 (12.3) | 36.9 (12.6) | 37.3 (11.9) | *>0.9 ^b^* |
| Body Mass Index (median) | 23.4 (3.7) | 22.7 (3.4) | 25.6 (4.6) | *0.016 ^b^* |
| Heart Variability: RMSSD (median) | 38 (30) | 40 (37) | 32 (17) | *0.2 ^b^* |
| RR-Interval (mean) | 887 (124) | 913 (125) | 830 (104) | *0.010 ^b^* |
| Depression: BDI-II (median) | 6 (10) | 3 (6) | 11 (12) | *<0.001 ^b^* |
| State Anxiety: STAI-S (median) | 31.0 (10.0) | 29.5 (9.3) | 32.0 (11.0) | *0.057 ^b^* |
| Trait Anxiety: STAI-T (mean) | 38.9 (10.3) | 37.4 (10.4) | 42.3 (9.6) | *0.084 ^b^* |
| Note: ^a^ = Chisq-test (count, %), ^b^ = t-test (mean, sd) | | | | |

At the level of trait, the group differences in the MAIA and the IAS would not remain as in the full population (p > 0.5976).

For the cluster analysis, the comparison between synch and asynch for HC showed a similar cluster to the main results (cluster 130–324ms, mainly centered over the FC1, C1, C2, CP1, and CPz electrodes), as well as an additional cluster occurring later (359–500ms, mainly centered over the CP3, CP1, CPz, P3, P1, and Pz electrodes). In the FND group, there was no significant difference compared to the main result. Crucially, when analyzing the between-group contrast, the effect is no longer significant. Consequently, this change in results might indicate a medication effect as a driver for the FND effect, or that this is simply due to the lower sample size (as this subsample analysis was done on half of the patients with FND while almost no HC had to be excluded due to the intake of medication).

Also on a global level, there are some differences in results when excluding medicated participants, with an overview illustrated in **Table S4**.

| **Table S4** | TANOVA sig. Time intervals | | GFP sig. Time intervals | |
| --- | --- | --- | --- | --- |
| Sample | full sample  (N = 78) | exclusion of medicated participants  (N = 59) | full sample  (N = 78) | exclusion of medicated participants  (N = 59) |
| Group Effect (FND vs HC) |  |  |  |  |
|  | 463-480ms | - | 10-20ms | - |
|  |  |  |  |  |
| Effect of Condition (synch vs asynch) |  |  |  | (-67) -(-50)ms |
|  |  |  | **31-55ms** | **28-55ms** |
|  | **56-72ms** | **59-72ms** | **77-96ms** | **70-106ms**  128-145ms |
|  | **144-167ms** | **145-158ms** |  |  |
|  | 382-410ms | **-** |  | 160-181ms |
|  |  |  | 247-261ms |  |
|  |  |  | **476-488ms** | **458-489ms** |
| Interaction Effect (Group*Condition) | (-17)-(-5)ms |  |  |  |
|  | **184-197ms** | 72-87ms  **182-197ms** |  |  |
|  | 215-241ms |  |  |  |
|  | 309-322ms |  | **360-380** | **364-381ms** |
|  |  | 390-408ms | **449-464** | **452-464ms** |
| Note: Overview of significant time intervals when running the global statistics with the 2x2 factor model, in the full sample (N = 78) or in the subsample (N = 59), with excluding participants that report an intake of medication (psychotropic, antiepileptic, opioid or benzodiazepine medication). Reported time intervals indicate a significant interval after R-peak in the omission period, with for p < 0.05 and a minimal duration of 10ms. Time periods that were robust for the medication effect are shown on the same line and are printed in bold font. | | | | |

For the effect of condition, we replicated most of the time intervals identified in the full population in the non-medicated participants as well. As apparent in Table S4, the interaction effects between condition and group of the full population were partially replicated for the TANOVA because only the result between 184-197ms was similar. The other periods did not match between the two analyses, even though they can be considered similar time intervals. In the GFP analysis, we observed the same interaction results. Again, we must consider that our identified group effect in the FND population might be driven by the medication, even though we cannot rule out that the disappearance of these results here might also be a power issue, because we had only half of the previous sample size to calculate with.

**References**

Cherneko, S. (2007). *ECG processing. R-peaks detection*. Librow: Kyiv. http://www.librow.com/articles/article-13

Garfinkel, S. N., Seth, A. K., Barrett, A. B., Suzuki, K., & Critchley, H. D. (2015). Knowing your own heart: Distinguishing interoceptive accuracy from interoceptive awareness. *Biological Psychology*, *104*, 65–74. https://doi.org/10.1016/j.biopsycho.2014.11.004

Petzschner, F. H., Weber, L. A., Wellstein, K. V., Paolini, G., Do, C. T., & Stephan, K. E. (2019). Focus of attention modulates the heartbeat evoked potential. *NeuroImage*, *186*, 595–606. https://doi.org/10.1016/j.neuroimage.2018.11.037

Pfeiffer, C., & De Lucia, M. (2017). Cardio-audio synchronization drives neural surprise response. *Scientific Reports*, *7*(1), Artikel 1. https://doi.org/10.1038/s41598-017-13861-8

Rangayyan, R. M. (2001). *Biomedical Signal Analysis*. IEEE. https://doi.org/10.1109/9780470544204

Stoffel, N., Sojka, P., Gninenko, N., Mouthon, M., von der Weid, L., Serranová, T., & Aybek, S. (2025). Respiratory sensitivity is reduced in functional neurological disorder and associated with higher somatoform dissociation. *Brain Communications*, fcaf283. https://doi.org/10.1093/braincomms/fcaf283
